# From the Breast to the Bowel: An Unconventional Metastatic Presentation

**DOI:** 10.7759/cureus.6199

**Published:** 2019-11-19

**Authors:** Mounika Gangireddy, Isha Shrimanker, Sandy Saintelia, Janet Gomez, Kathryn A Peroutka

**Affiliations:** 1 Internal Medicine, UPMC Pinnacle, Harrisburg, USA; 2 Hematology-Oncology, Andrews and Patel Associates, Harrisburg, USA

**Keywords:** breast cancer, metastasis, small bowel obstruction, iron deficiency anemia, gastrointestinal tract

## Abstract

Breast cancer is the most common cancer in women. The common sites of metastasis include the lungs, liver, and, infrequently, the gastrointestinal (GI) tract. A 72-year-old Caucasian female presented to the hospital with nausea and vomiting, diarrhea, intermittent abdominal pain, and unintentional weight loss. She had had a past medical history of bilateral lobular breast carcinoma and severe iron-deficiency anemia treated with iron transfusions. On arrival, the examination was significant for hypotension and pallor. Laboratory investigations revealed abnormal liver enzymes and raised tumor markers Ca-125 and carcinoembryonic antigen. Imaging studies established a diagnosis of distal small bowel obstruction. The surgical intervention showed the presence of a small bowel tumor, the biopsy findings of which were consistent with metastatic breast cancer, with ER and PR positive but HER-2 negative. She was managed with a selective estrogen receptor degrader and CDK4/6 inhibitor and has been in remission since. Metastasis to the small bowel from the breast is a very rare occurrence. Clinicians should thus maintain a modest amount of suspicion when encountering an uncommon GI presentation of primary breast malignancy. We describe the case of metastatic breast cancer with an atypical GI presentation.

## Introduction

Breast cancer accounts for 30% of all new cancer cases in women each year in the US, and it has a yearly mortality rate of 14% [1]. Among invasive breast carcinomas, invasive lobular cancer forms the second most common type with an incidence rate of 5-15% [2]. Invasive lobular carcinomas are characteristically multifocal when present in the unilateral breast, but more often they are present bilaterally [3].

Out of the diagnosed breast cancers, about 5-10% leads to metastasis, and at least 20-50% of patients develop metastasis at least once in their lifetime [4]. There has been a drastic increase in the survival rate of patients with breast cancer owing to timely recognition and prompt management. However, metastasis still occurs in 30% of the patients even after treatment with hormonal therapy, chemotherapy, radiotherapy, and surgical intervention [5]. Prognostic factors include the size and grade of the primary diagnosis, regional lymph node involvement, presence of hormonal receptors, and metastatic site involvement [6]. Breast cancer frequently metastasizes to the liver, lungs, brain, adrenals, and, very rarely, the bones. Metastasis to the gastrointestinal (GI) tract is atypical, although involvement can occur anywhere from the oropharynx to the anus [7]. We describe the case of an elderly female with GI metastasis, masked as small bowel obstruction, from primary breast cancer.

## Case presentation

A 72-year-old Caucasian female with a past medical history of bilateral lobular breast carcinoma, paroxysmal atrial fibrillation, and hyperthyroidism presented with an intermittent history of nausea and vomiting leading to decreased oral intake, diarrhea, intermittent abdominal pain, and unintentional weight loss of approximately 30-40 lb. The patient complained of an average of three bowel movements per day, more diarrhea than constipation with no change in consistency and absence of blood and mucus along with generalized abdominal pain, which was cramping in nature and got aggravated after meals. She denied heartburn or consuming any particular food that caused nausea.

The patient had been diagnosed with lobular carcinoma of the right breast 24 years ago. She had been managed with partial mastectomy, radiation, and adjuvant chemotherapy including fluorouracil, methotrexate, and cyclophosphamide followed by nine years of hormonal therapy with tamoxifen. The patient had been further diagnosed with lobular carcinoma of the left breast approximately 10 years after the initial diagnosis. She had undergone lumpectomy and had completed a five-year course of hormone-based chemotherapy with anastrozole. The patient had since been in remission.

She had had similar complaints of abdominal pain six months prior to her initial presentation. Workup at that time had revealed a hemoglobin of 6.5 gm/dl (normal range: 11.7-15.1 gm/dl), hematocrit of 21% (normal range: 29.4-47.0%), mean corpuscular volume (MCV) of 88 fl (normal range: 78.9-98.6 fl), and positive fecal occult blood. Iron studies indicated iron of 10 mcg/dl (normal range: 50-212 mcg/dl), a total iron-binding capacity of 381 mcg/dl (normal range: 250-450 mcg/dl), transferrin saturation of 3% (normal range: 15-50%) and ferritin of 6.8 ng/ml (normal range: 11-307 ng/ml). Imaging studies including CT of the abdomen had been unremarkable for any acute abnormality. Esophagogastroduodenoscopy (EGD) and colonoscopy showed mild gastritis and ulcer in the sigmoid colon along with internal and external hemorrhoids. The biopsy of the gastric ulcer had revealed no malignant changes. She had been managed with proton-pump inhibitor and blood transfusion. The patient had received parenteral iron transfusions as an outpatient.

**Figure 1:**
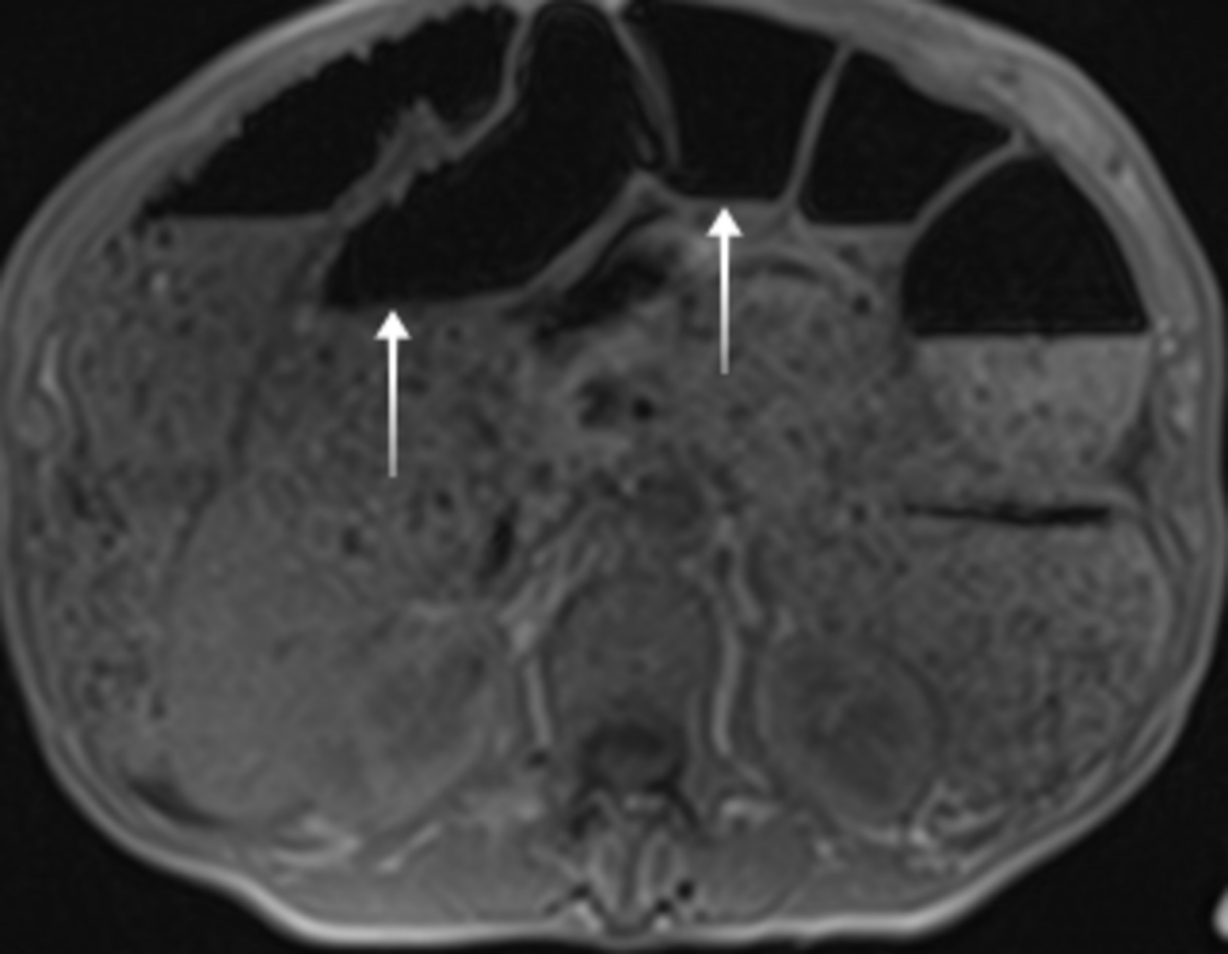
MRI of the abdomen showing distention of large intestine and small bowel with air-fluid levels concerning for distal obstruction. MRI: magnetic resonance imaging

**Figure 2:**
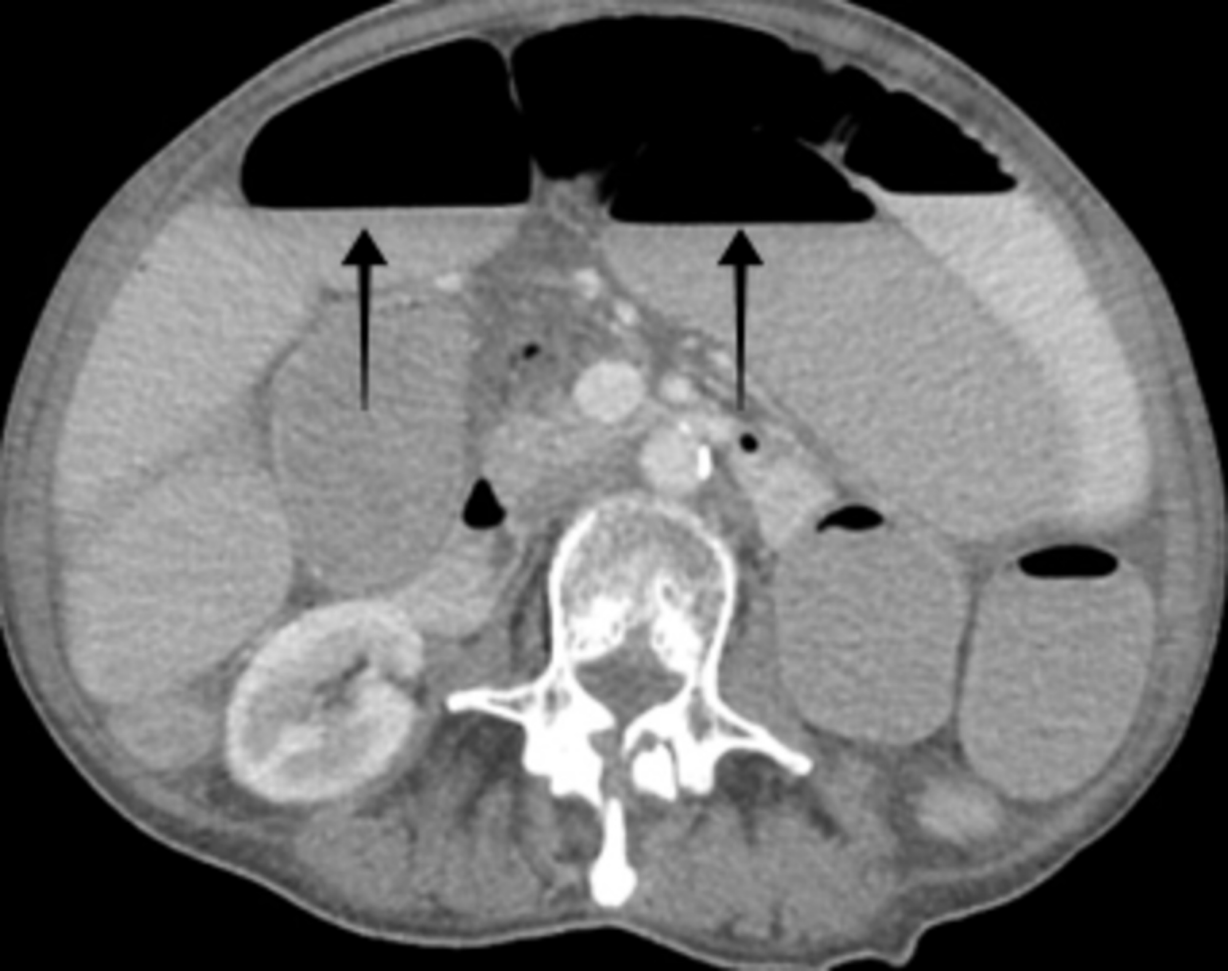
CT scan of the abdomen revealing high-grade small bowel obstruction with transition point noted centrally in the pelvis in the region of thickened small bowel loops. CT: computed tomography

On arrival to the emergency department, she had a pulse of 58 beats/minute, blood pressure of 98/46 mm Hg, oxygen saturation of 99% on room air, and body mass index of 19.5 kg/m^2^. A physical examination showed pallor. Abdominal examination was significant for distension along with tenderness in the left lower quadrant. Laboratory investigations were noteworthy for hemoglobin of 8.2 gm/dl, hematocrit of 24%, MCV of 89 fl, red cell distribution width of 16.8% (normal range: 11.5-15.5%). The hepatic panel revealed aspartate aminotransferase of 190 u/l (normal range: 13-39 u/l), alanine transaminase of 157 u/l (normal range: 7-52 u/l), and alkaline phosphatase of 27 u/l (normal range: 34-104 u/l). Further workup revealed Ca-125 of 93.6 u/ml (normal range: 0-35 u/ml) and carcinoembryonic antigen (CEA) of 12.6 u/ml (normal range: 0.0-3.0 u/ml). Imaging studies of the abdomen including radiograph, MRI with contrast (Figure 1), and CT scan (Figure 2) confirmed the presence of distal small bowel obstruction. The patient underwent small-bowel resection along with primary anastomosis after the detection of a small bowel tumor. 

**Figure 3:**
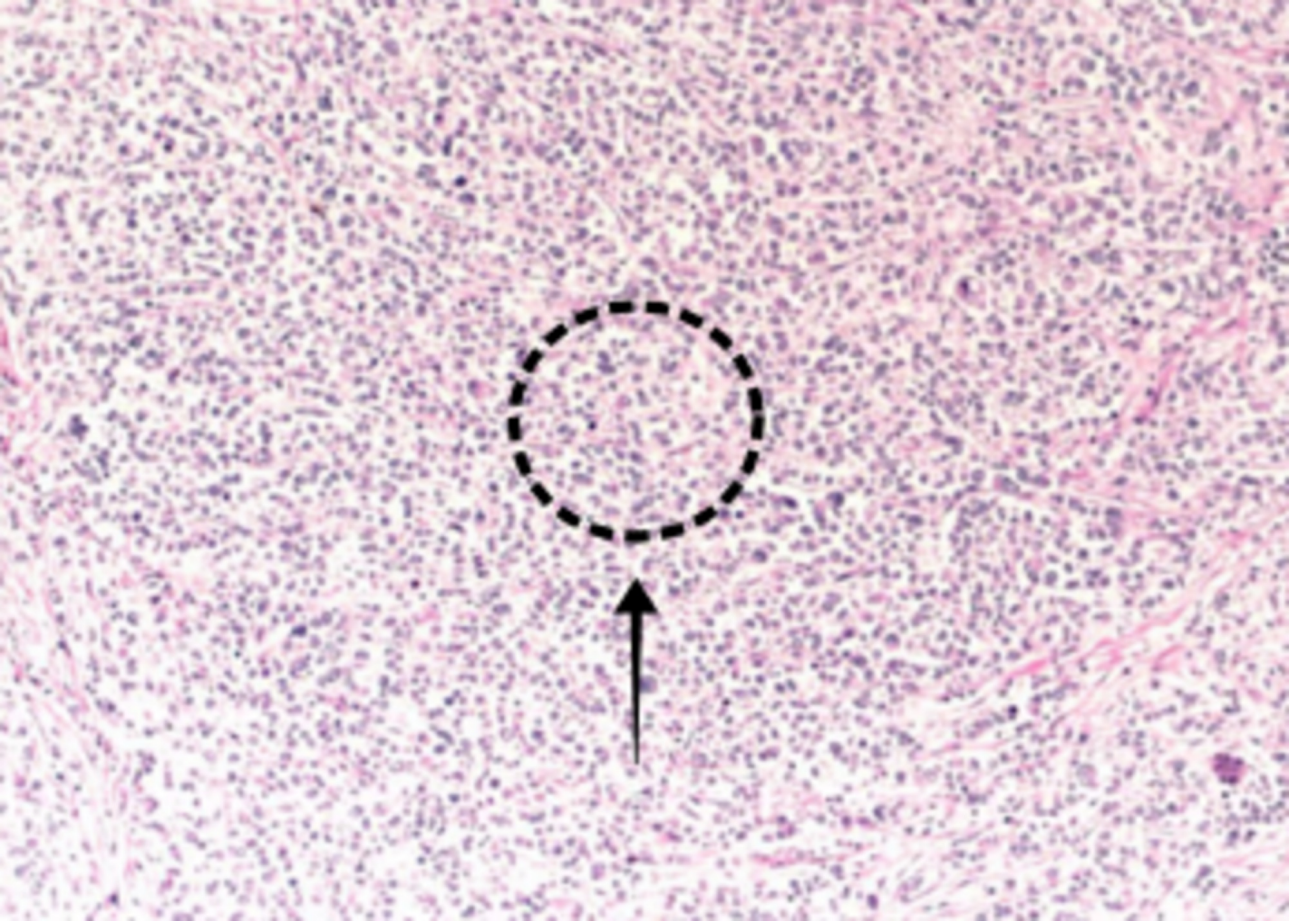
Biopsy specimen of resected small bowel showing multifocal metastatic pleomorphic lobular carcinoma present, consistent with breast primary. It shows positive mesenteric margin and negative proximal and distal surgical resection margins.

Histopathology showed the presence of metastatic pleomorphic lobular carcinoma consistent with breast primary (Figure 3). 

**Figure 4:**
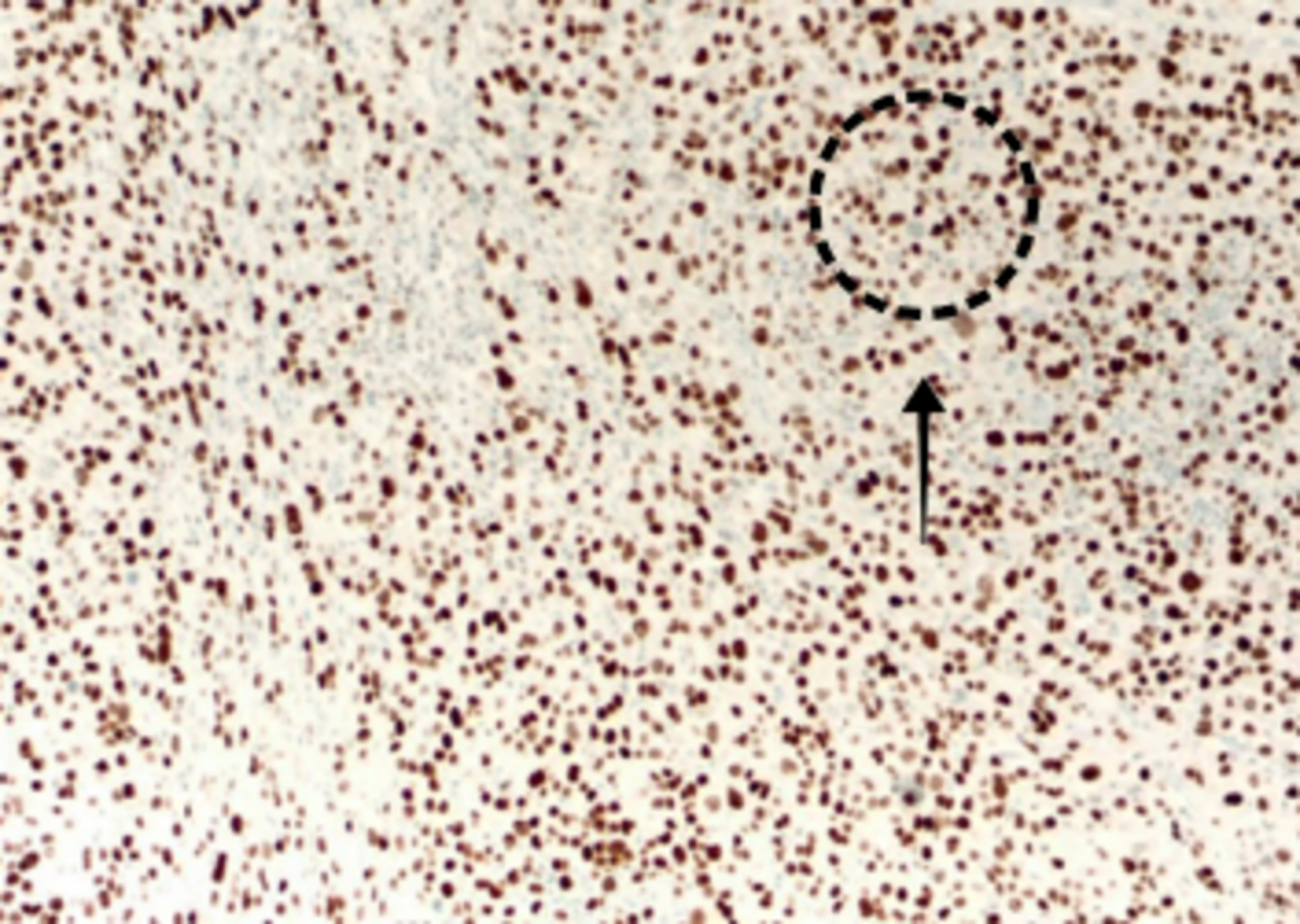
GATA 3+ stain. GATA3: GATA-binding protein 3

Further workup showed cytoplasmic staining with p120 catenin and positive for GATA-binding protein 3 (GATA-3) (Figure 4), focally positive for both estrogen receptor (ER) and progesterone receptor (PR), gross cystic disease fluid protein 15 (GCDFP-15) and mammaglobin, but negative for human epidermal growth factor receptor 2 (HER-2). 

**Figure 5:**
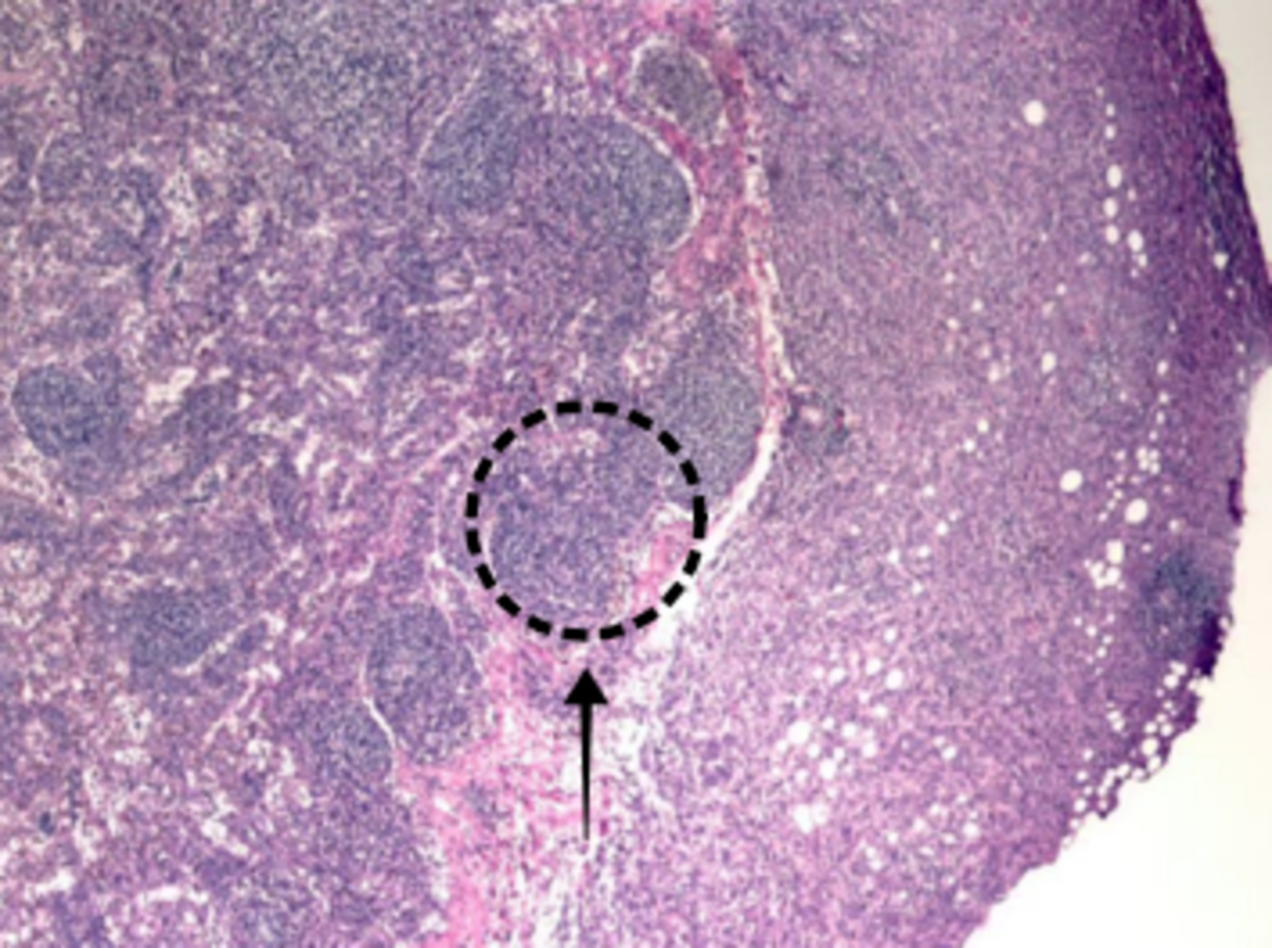
Lymph node positive for metastatic carcinoma.

Two lymph nodes near the tumor site were identified, out of which one demonstrated the presence of metastatic carcinoma (Figure 5). 

The patient was subsequently started on fulvestrant and palbociclib. She has shown significant improvement in her symptoms.

## Discussion

Small bowel malignancies comprise about 1-3% of primary GI cancers [8]. Small bowel metastasis is commonly seen in primary malignancies like colon cancer, malignant melanoma, and cervical cancer [9]. The involvement of the GI tract in primary breast cancer is a rare occurrence. Borst et al. reported that out of 2,500 cases of metastatic breast cancer they studied, only 17 cases (less than 1%) were found to have GI metastasis [10]. Stomach and small bowel metastasis are seen more often when compared to metastasis to the colon and rectum.

Lobular breast cancer metastasizes in a particular pattern and more commonly involves the GI tract and the retroperitoneum, even though infiltrative ductal breast carcinoma is more prevalent [11]. Invasive lobular cancer occurs more commonly in postmenopausal females, postulated to be due to hormone replacement therapy [12]. Our patient presented with bilateral lobular breast carcinoma that had metastasized to the small bowel and manifested with symptoms of obstruction.

Primary GI tumors are commonly positive for cytokeratin (CK) 20 and CEA, which is not seen in breast cancers. On the other hand, the metastatic lobular carcinoma is routinely positive for ER, PR, CK 7, and GCDFP-15 whereas it is negative for vimentin [13,14]. PR-positive breast cancers have a greater predisposition of GI metastasis when compared to ER-positive tumors [15]. Our patient was positive for CEA, ER and PR, and GCDFP-15, but negative for HER-2 tumor markers.

Previous studies have shown varying time durations between the diagnosis of primary breast cancer and the detection of GI metastasis. McLemore et al. revealed that the mean time interval between primary breast cancer and manifestations of metastatic cancer was seven years, with the median age of primary diagnosis being 55 years [16]. However, a literature search yielded an incidence of gastric metastasis 30 years after the primary diagnosis of breast cancer [17]. The present case shows GI metastasis after 24 years of the diagnosis of primary cancer.

The presentation of GI metastases ranges from no symptoms to manifestations of serious obstruction. As reported by McLemore et al., the frequent manifestations were abdominal pain, melena, nausea and vomiting, difficulty in swallowing, fatigue, weight loss, and a palpable mass [16]. Our patient presented with intermittent abdominal pain, unintentional weight loss, and severe iron-deficiency anemia.

There is no consensus in the medical fraternity about the management of breast cancer metastasizing to the GI tract. Several treatment modalities including systemic chemotherapy, hormonal therapy, and surgical procedure are available. Symptoms like intestinal obstruction, hemorrhage, or perforation necessitate surgical involvement. McLemore et al. reported in their study that GI metastatic patients who underwent palliative surgery had a longer median survival (44 months vs. nine months); however, it was statistically insignificant [16]. The study revealed that the average survival of metastasis to the GI tract from primary breast cancer was 28 months, with chemotherapy and tamoxifen treatment having a significant influence [16]. Tang et al. studied about two patients with metastatic lobular cancer presenting as intestinal obstruction who were treated with fulvestrant, a parenteral estrogen receptor antagonist [18]. Both cases showed the resolution of intestinal manifestations after fulvestrant administration. Our patient underwent resection of the small bowel tumor and was treated with selective estrogen receptor degrader, fulvestrant, and the CDK4/6 inhibitor, palbociclib.

## Conclusions

Instances of patients with primary breast cancer presenting with GI metastasis are uncommon and peculiar. These circumstances illustrate the importance of keeping GI metastasis as a likely differential, and physicians should be vigilant about patients with breast cancer, especially of the lobular type, who present with unusual GI manifestations.
